# ATF7–PINK1 Axis Governs Mitophagy and Intestinal Inflammation in Ulcerative Colitis

**DOI:** 10.1096/fj.202500813R

**Published:** 2025-06-30

**Authors:** Yidong Chen, Xiaopeng Zhang, Junrong Li, Fang Liu, Qi Yu, Jiamin Li, Liangru Zhu

**Affiliations:** ^1^ Division of Gastroenterology, Union Hospital, Tongji Medical College Huazhong University of Science and Technology Wuhan China; ^2^ Department of Endoscopy and Digestive System Guizhou Provincial People's Hospital Guiyang China; ^3^ Department of Gastroenterology The First Affiliated Hospital of Shihezi University Shihezi China

**Keywords:** ATF7, mitophagy, PINK1, ulcerative colitis

## Abstract

Ulcerative colitis (UC), a chronic inflammatory bowel disease, is marked by sustained inflammation and excessive apoptosis of intestinal epithelial cells (IECs). Despite progress in understanding UC pathogenesis, the role of activating transcription factors (ATFs) in disease progression remains elusive. Here, we profile the expression of ATF family members (ATF1–ATF7) in the colonic mucosa of UC patients and identify ATF7 as a critical regulator of mitophagy through its control of PTEN‐induced kinase 1 (PINK1). Expression levels of ATF1–ATF7 were quantified in colonic mucosal samples from UC patients (*n* = 219) and healthy controls (*n* = 105) via quantitative PCR. Using IEC‐specific ATF7 knockout mouse models and human CCD 841 CoN colonic epithelial cells, we employed ChIP‐seq, dual‐luciferase assays, transmission electron microscopy, and immunofluorescence to elucidate their roles in mitophagy and disease progression. Clinical correlation between ATF7 expression and disease severity was assessed using the Mayo score. ATF7 expression was significantly reduced in UC patients and inversely correlated with disease severity. Mechanistically, ATF7 was identified as a direct transcriptional activator of PINK1, a key mitophagy regulator. Loss of ATF7 or PINK1 disrupted mitophagy, exacerbating mitochondrial dysfunction, IEC apoptosis, and colonic inflammation in vivo and in vitro. Our findings uncover a pivotal ATF7‐PINK1 axis that governs mitophagy and limits UC progression. The inverse correlation between ATF7 expression and UC severity highlights its potential as a therapeutic target, offering new avenues for intervention in this debilitating disease.

## Graphical abstract

Ulcerative colitis is associated with diminished ATF7 expression in intestinal epithelial cells, which compromises PINK1 transcriptional activation and leads to impaired mitophagy, mitochondrial dysfunction, and excessive reactive oxygen species (ROS) accumulation. This cascade promotes epithelial injury and amplifies inflammation. Our findings identify the ATF7–PINK1 axis as a key regulator of mitochondrial homeostasis and a promising therapeutic target in UC.

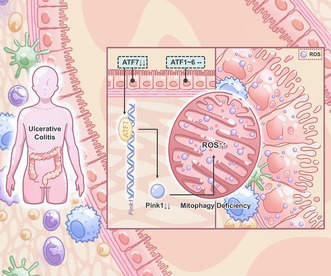

## 1 Introduction

Ulcerative colitis (UC) is a chronic inflammatory bowel disease marked by a distinct pathology that includes superficial ulcerations, inflammatory infiltration, and increased apoptosis of intestinal epithelial cells (IECs) [1, 2, 3]. These features contribute significantly to the disease's progression, with the recurrent inflammation in the colonic mucosa leading to a globally increasing incidence [4]. The enhanced apoptosis of IECs, coupled with inflammation and mucosal ulceration, plays a pivotal role in exacerbating UC and complicating the healing process of the colonic tissue.

IECs, forming the innermost layer of the gut, are pivotal in nutrient absorption, maintenance of intestinal barrier functions, and regulation of the host immune system [5]. They provide a barrier against pathogenic microorganisms and harmful substances while permitting the passage of beneficial microbes and nutrients [6, 7]. Through interactions with various components of the intestinal immune system, IECs modulate local immune responses and have been postulated to serve as core regulators of intestinal immune homeostasis [8]. Consequently, strategies aimed at mitigating inflammatory responses in IECs, maintaining the balance of IEC proliferation and apoptosis, and preserving intestinal epithelial integrity have become crucial in UC treatment.

Mitochondria, essential cellular powerhouses for homeostasis, play central roles in ATP production through oxidative phosphorylation, emphasizing their involvement in energy metabolism [9, 10]. They also serve as central nodes for signal transduction and intermittently produce reactive oxygen species (ROS) via the electron transport chain [11, 12]. Excessive ROS can induce oxidative stress, resulting in cellular dysfunction, aging, and various pathologies [13, 14]. Moreover, mitochondria meticulously maintain cellular redox balance, and any dysregulation can disrupt this equilibrium, leading to oxidative stress, cellular damage, and pathological conditions [15, 16]. Under severe stress, mitochondria regulate apoptosis by releasing cytochrome c, triggering cell death [17, 18]. Thus, the multifaceted roles of mitochondria in energy production, signal transduction, redox balance, and apoptosis underscore their criticality to cellular function, survival, and disease pathogenesis [19].

Mitophagy, a pivotal process for mitochondrial quality control and cellular homeostasis [20], is orchestrated by multiple pathways [21, 22]. The BNIP3/BNIP3L pathway contributes to the encapsulation of damaged mitochondria into autophagosomes [23, 24]. The PINK1/Parkin pathway plays a crucial role in mitochondrial surveillance. It involves PTEN‐induced kinase 1 (PINK1) identifying and marking impaired mitochondria, subsequently recruiting Parkin, which ubiquitinates mitochondrial proteins [25, 26]. These ubiquitinated proteins are recognized by autophagy receptors, linking the mitochondria to the autophagic machinery. Additionally, the FUNDC1 pathway mediates the autophagic removal of dysfunctional mitochondria under hypoxic conditions [27, 28]. These mechanisms collectively enhance cellular resilience to stress‐induced apoptosis. Importantly, the detection and quantification of mitophagy should be standardized and performed in accordance with the most recent guidelines for autophagy assays [29].

The PINK1/Parkin pathway, in particular, involves complex molecular interactions. Upon phosphorylation and activation by PINK1 at Ser65, Parkin (PRKN) modifies various outer mitochondrial membrane proteins, mainly with K11‐ and K63‐linked ubiquitin chains [30]. This modification enhances the pool of phospho‐ubiquitin, which recruits autophagy receptors such as SQSTM1/p62, OPTN, and MAP1LC3/LC3 to the damaged mitochondria [31, 32]. This process culminates in the removal of these mitochondria via mitophagy. The ultimate fate of mitochondria undergoing mitophagy is their movement towards lysosomes for degradation [33, 34]. However, the intricate roles and mechanisms of PINK1/Parkin‐mediated mitophagy, particularly in the context of IEC apoptosis and UC pathogenesis, remain to be fully explored, indicating a significant area for future research.

Within the Activating Transcription Factor (ATF)/cyclic AMP‐responsive element‐binding (CREB) family of transcription factors, several members have been extensively studied, highlighting their pivotal roles in processes such as aging and inflammation [35, 36]. ATF1 has been implicated in the phenotypic switching of macrophages, thereby contributing to the progression of inflammation [37]. ATF2 is closely associated with epithelial cell homeostasis and plays a critical role in maintaining tissue integrity [38]. Studies have demonstrated that ATF3 alleviates intestinal inflammation by inhibiting the maturation of mononuclear macrophages [39]. Among the family members, ATF4 has been the most extensively investigated; its deficiency exacerbates intestinal inflammation in mice by reducing glutamine uptake and suppressing the expression of antimicrobial peptides [40]. ATF5 is believed to participate in the regulation of downstream inflammatory factors via the NF‐κB pathway [41]. ATF6, in contrast, facilitates mitophagy by upregulating MIR346, which enhances the translation of GSK3B and promotes the dissociation of BCL2 and BECN1. This process supports the clearance of damaged mitochondria, reduces reactive oxygen species (ROS) levels, and protects cells from oxidative stress‐induced damage [42].

However, compared to its well‐characterized counterparts, ATF7 remains relatively underexplored. Despite the pivotal roles played by ATF family members in cellular stress responses, differentiation, growth, and immune modulation, ATF7's unique contributions and mechanisms have not been thoroughly delineated [36, 43]. Our investigation positions ATF7 at the forefront of our inquiry into mitochondrial health and apoptosis in IECs. Preliminary findings suggest that ATF7 may critically influence PINK1 expression and, consequently, mitophagy—a vital process for the clearance of damaged mitochondria. This positions ATF7 as a potential key player in the intricate nexus between mitochondrial function, cellular apoptosis, and the pathogenesis of conditions such as UC, marking a novel frontier in our understanding of cellular physiology and disease mechanisms.

In addition to its known roles in cellular stress response, differentiation, and growth, ATF7 has been reported to be intricately involved in the repair of intestinal epithelial injury [44]. Although the specific mechanisms are not yet elucidated, this newly identified function further complicates our understanding of ATF7's multifaceted impact on cellular physiology. Given the chronic inflammation and epithelial damage that are hallmarks of ulcerative colitis, this opens new avenues for future research into the broader roles that ATF7 might play in gastrointestinal health and disease.

Our study delves into the intricate interplay between mitophagy, apoptosis, and the pathogenesis of UC through a multifaceted approach. Utilizing techniques such as ChIP‐seq, targeted gene knockout in murine intestinal epithelial cells, flow cytometry, and advanced in vitro cell culture, we have uncovered a novel regulatory role for ATF7 in modulating PINK1 expression—an essential mediator of mitophagy in IECs. This ATF7‐PINK1 axis sheds light on critical molecular mechanisms underlying UC and opens new avenues for targeted therapeutic interventions. Our ongoing research aims to further elucidate this regulatory pathway, with promising implications for mitigating inflammation and apoptosis in UC, ultimately paving the way for novel therapeutic strategies.

## 2 Methods

### 2.1 Reagents and Antibodies

For flow cytometry, the Mitochondrial Membrane Potential Detection Kit (BD Biosciences, Cat# 551302, NJ, USA), Annexin V Apoptosis Detection Kit I (BD Biosciences, Cat# 556547, NJ, USA), and 2′,7′‐Dichlorofluorescein diacetate (Sigma‐Aldrich, Cat# 35845, MO, USA) were utilized.

Chromatin immunoprecipitation assays were conducted using the Simple ChIP Plus Enzymatic Chromatin IP Kit (Cell Signaling Technology, Cat# 9004, MA, USA) along with anti‐ATF7 antibody (Abcam, Cat# ab183507, Cambridge, UK). Quantitative PCR was performed with SYBR Green PCR Master Mix (Takara, Cat# RR036A, Shiga, Japan).

For ELISA assays, kits for IL‐10 (Proteintech, Cat# KE10008, Wuhan, China) and IL‐1β (Proteintech, Cat# KE00021, Wuhan, China), as well as TNF‐α (Beyotime, Cat# PT513, Shanghai, China) and IL‐17 (Beyotime, Cat# PI545, Shanghai, China) were used.

Antibodies for Western blot and immunofluorescence staining in Table S1.

Dextran sulfate sodium (DSS) for colitis induction was obtained from MP Biomedicals (Cat# 9011, CA, USA), and the luciferase reporter vector pGL4.11 was sourced from Promega (WI, USA) for transfection experiments.

### 2.2 Patient Tissue Sampling

This investigation involved tissue specimens from 324 individuals treated at Wuhan Union Hospital from September 2020 to October 2023. The cohort comprised 219 UC patients in the active phase and 105 control individuals with non‐inflammatory, non‐neoplastic conditions, all undergoing colonoscopic examinations. UC patients were stratified based on clinical symptoms (abdominal pain, diarrhea, mucopurulent bloody stool) and Mayo endoscopic subscore (MES): 107 patients with MES of 1 and another 112 with MES of 2–3. Biopsy samples from UC patients were specifically harvested from inflamed rectal sites, mirroring the control sample collection site. Each sample was bifurcated: one part was fixed in paraformaldehyde for ensuing qPCR, the other preserved for ChIP‐seq. qPCR‐designated samples were instantly snap‐frozen and stored at −80°C. Informed consent was scrupulously acquired from all subjects before sampling. The study protocol, including sample collection, received approval and oversight from the Independent Ethics Committee of Wuhan Union Hospital. Patient demographics and clinical data were meticulously documented, as detailed in Table 1.

**TABLE 1:** Demographic and clinical profiles of study participants at Wuhan Union Hospital from September 2020 to October 2022.

Patient characteristics	UC_Mayo ES 1	UC_ Mayo ES 2–3	Control
**Patients (*n*)**	107	112	105
Female	53	51	54
Male	54	61	51
Age (mean ± SD)/year	27.31 ± 5.23	25.94 ± 6.02	30.75 ± 4.75
Age range/year	18–43	18–44	18–45
**Mayo ES**			
Mayo Score 0	0	0	NA
Mayo Score 1	107	0	NA
Mayo Score 2	0	59	NA
Mayo Score 3	0	53	NA
**Biopsy site**			
Rectum	107	112	105

### 2.3 Animal Models and Experimental Design

Wild‐type (WT) C57BL/6J mice were sourced from Cavens, Changzhou, China. To generate intestinal epithelial cell‐specific ATF7 knockout models, ATF7^flox/flox^ mice were acquired from Modelorg, Shanghai, China. These mice were then crossbred with Villin‐Cre mice, sourced from The Jackson Laboratory, Bar Harbor, ME, USA, to specifically delete ATF7 in intestinal epithelial cells, hereafter referred to as *ATF7* ^−/−^. The Villin‐Cre and flox/flox mice were all on a C57BL/6J genetic background and engineered using the CRISPR/Cas9 system. For each experimental condition, cohorts comprised six mice per group. No data were excluded from the analysis in this study.

Animals were housed under regulated conditions with ad libitum access to standard chow and water. Acute colitis was induced through the administration of 2.5% DSS (w/v) in the drinking water, sustained over an 8‐day course. On the eighth day following DSS administration, the mice were humanely euthanized, and their colonic tissues were harvested. A segment measuring 0.5 cm from the distal colon was meticulously reserved for histopathological analyses, including Hematoxylin & Eosin (H&E) staining, immunofluorescence microscopy, and transmission electron microscopy, the latter specifically for mitochondrial examination. The conduct of all animal experiments was in strict alignment with the ethical guidelines and approvals provided by the Institutional Research Ethics Committee of Tongji Medical College, Huazhong University of Science and Technology.

### 2.4 ChIP‐Seq Analysis

Intestinal mucosal samples from UC patients were utilized for chromatin immunoprecipitation sequencing (ChIP‐seq) assays. The process commenced with cell cross‐linking, followed by the extraction of nuclear lysates. Post‐sonication, chromatin fragments were immunoprecipitated using monoclonal antibodies against ATF7. The precipitated chromatin complexes were then subjected to deep sequencing at Frasergen, Wuhan, China. In the analytical phase, raw sequencing data were meticulously filtered to obtain high‐quality sequences. These refined sequences were aligned against the human reference genome (hg38), facilitating comprehensive peak calling across the genome.

### 2.5 Transmission Electron Microscopy (TEM)

For the visualization of mitochondria, mouse colonic tissues were prepared for TEM. Briefly, tissue samples were initially fixed with 2.5% glutaraldehyde in 0.1 M phosphate buffer (pH 7.4) at 4°C overnight, and subsequently post‐fixed with 1% osmium tetroxide. After the fixation process, the specimens underwent dehydration through a graduated series of ethanol concentrations, followed by a transition into propylene oxide before being embedded in Epon 812. Sections of approximately 70 nm in thickness were obtained using an ultramicrotome, placed on copper mesh grids, and subsequently stained with both uranyl acetate and lead citrate. The specimens were then examined under a Hitachi HT7800 transmission electron microscope (Hitachi, Tokyo, Japan) operating at an accelerating voltage of 80 kV. Micrographs were taken, and mitochondria in the IECs were analyzed.

### 2.6 Histological Staining

Histological analysis was performed on distal colon sections (0.5 cm) collected from euthanized mice. Tissues were fixed in 10% neutral‐buffered formalin overnight at room temperature, embedded in paraffin, and sectioned into 5‐μm slices. Sections were stained with hematoxylin and eosin (H&E) using standard protocols, where hematoxylin highlighted nuclear features and eosin served as a counterstain for cytoplasmic and extracellular matrix structures. Histological scoring was conducted independently by two blinded pathologists, assessing inflammation severity, tissue damage, and inflammatory cell infiltration; discrepancies were resolved by consensus. Superoxide production was evaluated using dihydroethidium (DHE) staining, sections were incubated with 5 μM DHE at 37°C for 30 min in a light‐protected, humidified chamber, followed by thorough washing and mounting with antifade medium. Fluorescence images were captured and analyzed by a blinded pathologist, with quantitative assessments performed across multiple representative fields per slide.

### 2.7 Cell Culture and Transfection

The human colonic epithelial cell line CCD841 CoN was obtained from the American Type Culture Collection (ATCC, Manassas, VA, USA) and cultured in accordance with the supplier's instructions. To generate ATF7‐deficient cells, specific single guide RNAs (sgRNAs) targeting ATF7 were synthesized and delivered by Genechem Co. Ltd. (Shanghai, China) using a CRISPR/Cas9‐mediated genome editing system. Cells were transfected with sgRNAs and Cas9‐expressing constructs using Lipofectamine 3000 (Thermo Fisher Scientific, MA, USA) according to the manufacturer's protocol. For re‐expression experiments, full‐length human ATF7 cDNA was cloned into the pcDNA3.1(+) expression vector and transiently transfected into ATF7‐deficient cells using Lipofectamine 3000. Cells transfected with an empty vector served as controls. To evaluate mitophagic flux, cells were treated with bafilomycin A1 (50 nM, Sigma‐Aldrich), a known inhibitor of autophagosome‐lysosome fusion, for 4 h prior to analysis.

### 2.8 ELISA Analysis

Cytokine concentrations in mouse colon homogenates were quantified using Enzyme‐Linked Immunosorbent Assays (ELISAs). Levels of key inflammatory mediators, including IL‐10, IL‐1β, TNF‐α, and IL‐17, were determined following the protocols provided by the respective ELISA kit manufacturers. Colon homogenates were thawed and processed according to the kit instructions, and absorbance was measured at 450 nm using an Enspire microplate reader (PerkinElmer, Waltham, MA, USA). Cytokine concentrations were calculated from standard curves, with all measurements performed in triplicate to ensure accuracy and reproducibility.

### 2.9 Quantitative Real‐Time PCR Analysis

Quantitative real‐time PCR (qPCR) was conducted using a Roche Real‐Time PCR Instrument (Roche Diagnostics, Indiana, USA). The reaction mixture consisted of complementary DNA (cDNA), gene‐specific primers (ATF1: Forward 5′‐AGGACTCATCCGACAGCATAG‐3′, Reverse 5′‐TTCTGCCCCGTGTATCTTCAG‐3′; ATF2: Forward 5′‐AATTGAGGAGCCTTCTGTTGTAG‐3′, Reverse 5′‐CATCACTGGTAGTAGACTCTGGG‐3′; ATF3: Forward 5′‐CCTCTGCGCTGGAATCAGTC‐3′, Reverse 5′‐TTCTTTCTCGTCGCCTCTTTTT‐3′; ATF4: Forward 5′‐ATGACCGAAATGAGCTTCCTG‐3′, Reverse 5′‐GCTGGAGAACCCATGAGGT‐3′; ATF5: Forward 5′‐TGGCTCGTAGACTATGGGAAA‐3′, Reverse 5′‐ATCAACTCGCTCAGTCATCCA‐3′; ATF6: Forward 5′‐TCCTCGGTCAGTGGACTCTTA‐3′, Reverse 5′‐CTTGGGCTGAATTGAAGGTTTTG‐3′; ATF7: Forward 5′‐GAGACGACAGACCGTTTGTGT‐3′, Reverse 5′‐AGGCGTTTGATCTGCAATGAT‐3′; GAPDH: Forward 5′‐GGAGCGAGATCCCTCCAAAAT‐3′, Reverse 5′‐GGCTGTTGTCATACTTCTCATGG‐3′), sourced from PrimerBank (https://pga.mgh.harvard.edu/primerbank/), and SYBR Green PCR Master Mix. Thermal cycling conditions included an initial denaturation at 95°C for 3 min, followed by 40 cycles of 95°C for 15 s, 60°C for 30 s, and 72°C for 30 s. Amplification specificity was confirmed through melting curve analysis. Each reaction was performed in triplicate to ensure consistency and reproducibility. Relative expression levels of target genes were calculated using the 2^^−ΔΔCT^ method and normalized to the expression of the housekeeping gene GAPDH.

### 2.10 Immunofluorescence Staining

For immunofluorescence studies on tissue samples, sections of mouse colon embedded in paraffin were first subjected to deparaffinization and rehydration, followed by antigen unmasking procedures. Thereafter, these sections were pretreated to block non‐specific binding and then incubated with primary antibodies at a 1:100 dilution, maintained at 4°C overnight. Post washing, slides were incubated with fluorescently labeled secondary antibodies.

For cellular immunofluorescence, CCD841 CoN cells were fixed, permeabilized, and incubated with primary antibodies (diluted 1:100) overnight at 4°C. After washing, cells were incubated with fluorescently labeled secondary antibodies. Nuclei were counterstained with DAPI. Live Cell Staining: For live cell imaging, CCD841 CoN cells were stained with LysoTracker Red (50 nM), MitoTracker Green (50 nM), or MitoTracker Red (500 nM), respectively, for 1 h at 37°C, and then treated with Hoechst stain to label nuclei. Unbound dye was removed by rinsing with fresh culture medium.

Mitophagy Assay Using mito‐Keima: To monitor mitophagy flux, CCD841 CoN cells were transfected with the mitochondrial‐targeted pH‐sensitive fluorescent protein mito‐Keima. Following transfection, cells were maintained under experimental conditions and then imaged using excitation at dual wavelengths (440 and 561 nm) to distinguish mitochondrial populations in neutral versus acidic environments, reflecting mitochondrial delivery to lysosomes.

Immunofluorescence imaging: After completing the staining procedures, both fixed and live samples were immediately examined using a Zeiss LSM‐800 confocal microscope (Carl Zeiss AG, Oberkochen, Germany).

### 2.11 Western Blotting

Proteins were extracted from IECs isolated from murine and human colonic tissues, using RIPA buffer (Thermo Fisher Scientific, MA, USA), supplemented with protease and phosphatase inhibitors (Roche, Basel, Switzerland). Protein concentrations were determined with a BCA Protein Assay Kit (Pierce, IL, USA). Equal amounts of protein (20–30 μg) were resolved on 10% or 12% SDS‐PAGE gels and transferred to PVDF membranes (Millipore, MA, USA).

Membranes were blocked with 5% non‐fat milk or BSA in TBS‐T for 1 h, followed by overnight incubation at 4°C with primary antibodies in TBS‐T containing 1% BSA. After washing, membranes were incubated with HRP‐conjugated secondary antibodies for 1 h. Protein bands were detected using an ECL system (GE Healthcare, NJ, USA) and quantified with ImageJ software (NIH, USA). Densitometry values were normalized to GAPDH as a loading control.

### 2.12 Chromatin Immunoprecipitation (ChIP) Assays

ChIP assays were performed to investigate the interaction between ATF7 and the PINK1 promoter in CCD 841 CoN cells. Following formaldehyde fixation, the cross‐linked protein‐DNA complexes were isolated using a commercial ChIP kit as per the manufacturer's instructions. The complexes were then subjected to an overnight incubation at 4°C with gentle agitation, in the presence of either an ATF7 primary antibody or rabbit IgG as a negative control. Post‐incubation, magnetic beads were introduced and incubated for 2 h with gentle agitation to facilitate the isolation of the antibody‐bound complexes. The bead‐bound DNA was subsequently eluted and subjected to PCR amplification using primers flanking the ATF7 binding site on the human PINK1 promoter. The primers used were 5′‐TGCCGCTGAAGCCAGAGACTA‐3′ (forward) and 5′‐CCCATAGCCAAGTAAGCCCAGG‐3′ (reverse).

### 2.13 Luciferase Reporter Assays

Luciferase reporter assays were performed using the pGL4.11‐Basic vector, engineered to contain either the wild‐type (WT) or mutant (Mut) promoter region of PINK1. ATF7‐knockdown and control cells were co‐transfected with these reporter constructs alongside the pRL Renilla luciferase vector as an internal control. Dual‐luciferase activities were measured using a luminometer (Promega, Madison, USA), allowing quantification of Firefly and Renilla luciferase signals within the same sample. Renilla luminescence was used to normalize Firefly luciferase activity, ensuring correction for transfection efficiency and variability in sample handling. Normalized Firefly luciferase activity provided a robust measure of promoter activity under each experimental condition.

### 2.14 Flow Cytometry Analysis

CCD841 CoN cells were subjected to flow cytometry analysis for the evaluation of apoptosis and determination of mitochondrial membrane potential (Δψm) and reactive oxygen species (ROS) production. The following markers were used:

Apoptosis: The cells were subjected to staining procedures using the Annexin V‐FITC/PI apoptosis detection kit, in accordance with the guidelines provided by the manufacturer. In summary, cells were reconstituted in binding buffer, to which Annexin V‐FITC and propidium iodide (PI) were subsequently added. Following a 15‐min incubation period in dark conditions, cellular analysis was conducted.

Δψm: JC‐1 staining was performed using the JC‐1 Mitochondrial Membrane Potential Assay Kit. Cells were incubated with JC‐1 dye at 37°C for 30 min, washed, and then subjected to flow cytometry. JC‐1 forms aggregates and emits at 590 nm (red) in healthy cells (high Δψm), while it remains in monomeric form, emitting at 527 nm (green) in apoptotic or unhealthy cells (low Δψm).

ROS production: Cells were stained with 2′,7′‐dichlorofluorescin diacetate (DCFH‐DA) for detection of intracellular ROS. After incubation with DCFH‐DA at 37°C for 30 min, cells were washed and resuspended for flow cytometry.

Flow cytometric analyses were performed using a BD FACSCalibur Flow Cytometer (BD Biosciences, San Jose, CA, USA). A minimum of 10 000 events were recorded for each sample. The collected data were analyzed using FlowJo software (Tree Star Inc., OR, USA).

### 2.15 Statistical Analysis

For our statistical analysis, we expressed all numerical data as mean ± standard deviation. When making single comparisons, we utilized an unpaired two‐tailed Student's *t*‐test, while for multiple comparisons, we employed a one‐way analysis of variance (ANOVA). In cases of non‐parametric distributions, we used the Mann–Whitney *U* test. We set a *p*‐value of less than 0.05 to denote statistical significance. We performed all statistical procedures using GraphPad Prism version 9.5 (GraphPad Software Inc., USA).

## 3 Results

### 3.1 Downregulation of ATF7 Correlates With Ulcerative Colitis Progression and Mitochondrial Dysfunction

To investigate the changes in ATF family transcription factors in ulcerative colitis (UC), we collected intestinal biopsy specimens from control individuals and UC patients. UC samples were categorized into two groups based on Mayo endoscopic subscore (Mayo ES): Mayo ES 1 and Mayo ES 2–3. The mRNA expression levels of all reported *ATF* family members were measured.

**FIGURE 1:**
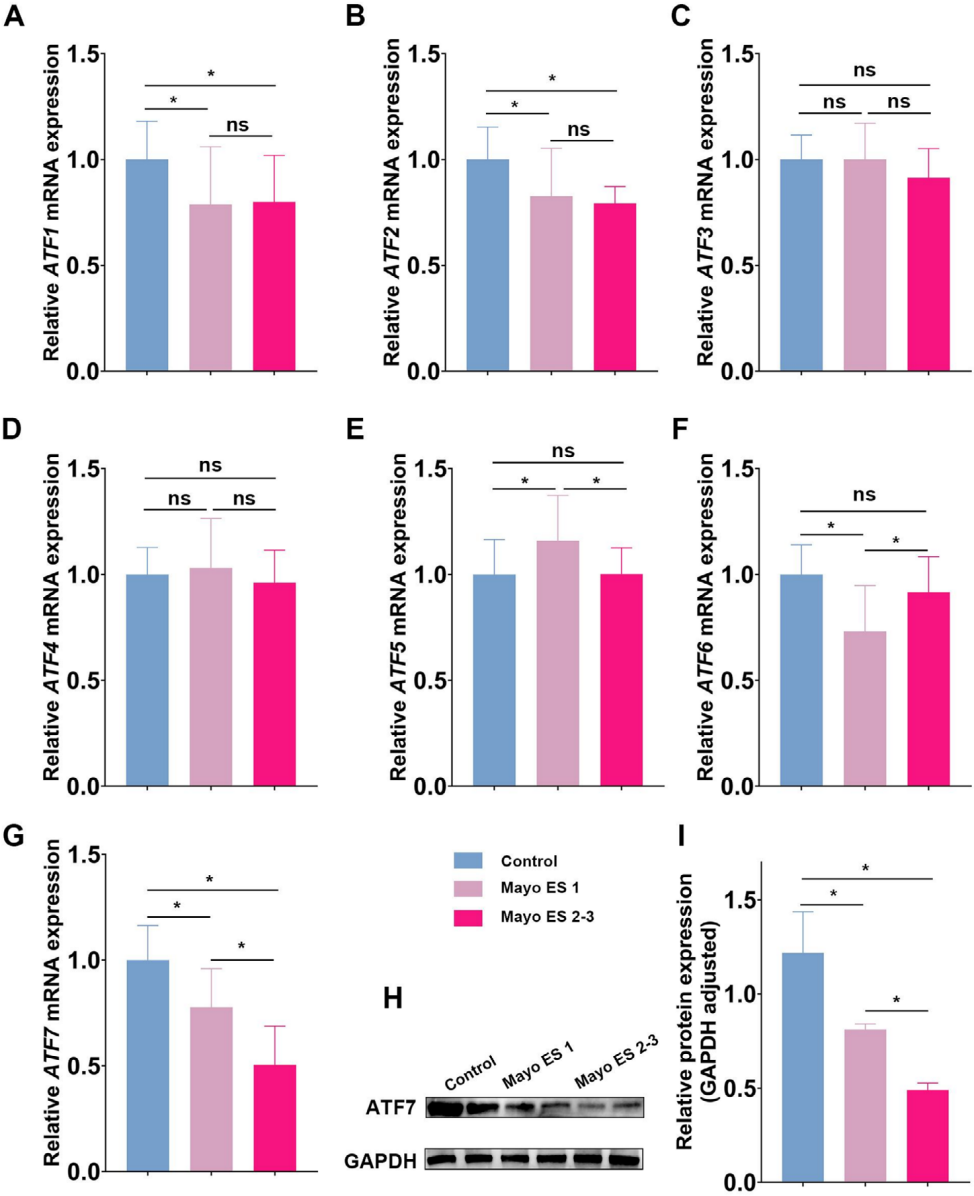
Differential expression of ATF family transcription factors in the colonic mucosa of UC patients. Relative mRNA expression levels of *ATF* family members (A–G, *ATF1*–*ATF7*) were assessed in intestinal biopsy specimens from control individuals (*n* = 105) and UC patients, who were stratified into two groups based on the Mayo endoscopic subscore: Mayo ES 1 (*n* = 107) and Mayo ES 2–3 (*n* = 112). Compared to controls, the Mayo ES 1 group exhibited significantly reduced expression of *ATF1* (A), *ATF2* (B), *ATF6* (F), and *ATF7* (G), whereas *ATF3* (C) and *ATF4* (D) showed no significant differences. In contrast, *ATF5* (E) expression was significantly elevated in the Mayo ES 1 group. Notably, among UC patients, ATF7 expression was lowest in the Mayo ES 2–3 group, indicating a correlation with disease severity (G). Gene expression levels were normalized to *GAPDH*. (H) Representative Western blot images of ATF7 and GAPDH protein expression in intestinal biopsy specimens. (I) Quantification of ATF7 protein levels (*n* = 6 per group), confirming consistency with mRNA expression patterns. Data are presented as mean ± SD. Statistical significance was determined by one‐way ANOVA (*p* < 0.05; ns, not significant).

In the Mayo ES 1 group, significant reductions in the expression of *ATF1*, *ATF2*, *ATF6*, and *ATF7* were observed compared to controls, while the expression levels of *ATF3* and *ATF4* showed no significant change. Interestingly, *ATF5* expression was elevated in the Mayo ES 1 group compared to controls (Figure 1). Notably, patients in the Mayo ES 2–3 group, representing more severe UC, exhibited the lowest levels of *ATF7* mRNA (Figure 1G). Western blot analysis of ATF7 expression confirmed that protein levels mirrored the mRNA findings (Figure 1H,I).

These results suggest that the expression of several ATF family members, particularly ATF7, is significantly altered in UC and that ATF7 expression progressively decreases with increasing disease severity. Overall, this highlights ATF7 as a potential biomarker for UC severity and provides novel insights into its regulation in the context of intestinal inflammation.

**FIGURE 2:**
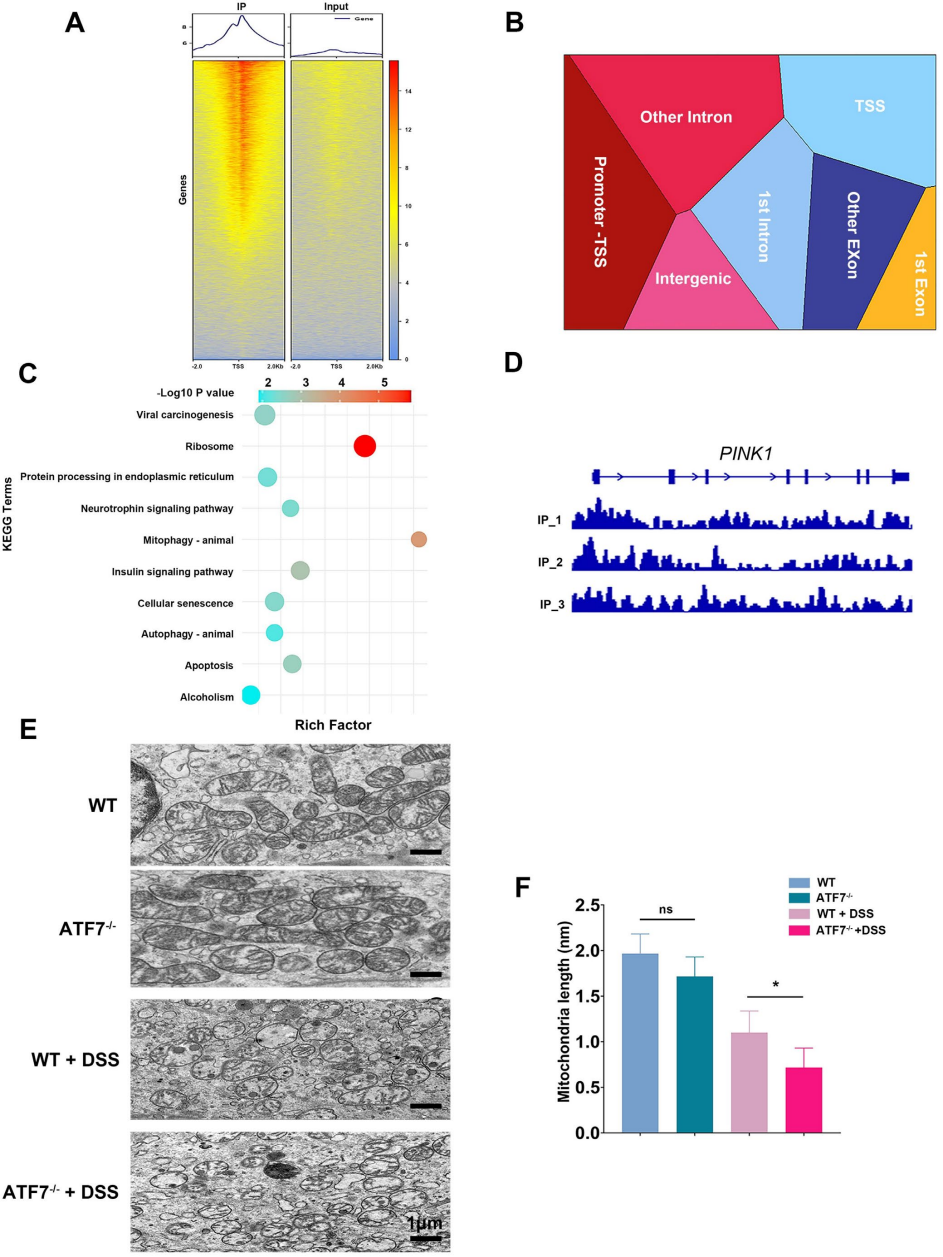
ATF7 regulates PINK1 expression and mitochondrial integrity in colonic epithelial cells. (A) Heatmap showing ChIP‐seq read distribution around transcription start sites (TSS) in colonic mucosal samples from UC patients. (B) Genomic annotation of enriched ChIP‐seq peaks indicates significant localization at TSS regions. (C) KEGG pathway enrichment analysis of genes associated with ChIP‐seq peaks reveals multiple pathways, with mitophagy displaying the highest rich factor. (D) Visualization of ChIP‐seq reads at the PINK1 transcription start site (TSS) using Integrative Genomics Viewer (IGV), highlighting ATF7 enrichment. (E) Transmission electron microscopy images of colonic epithelial cells from wild‐type (WT) mice and ATF7‐deficient (*ATF7* ^−/−^) mice, with or without DSS‐induced colitis. Damaged mitochondria, characterized by swelling, were more prevalent in the *ATF7* ^−/−^ + DSS group. Scale bar: 1 μm. (F) Quantification of mitochondrial length, indicating a reduction in the *ATF7* ^−/−^ + DSS group. Data are presented as mean ± SD. Statistical significance was determined using one‐way ANOVA (*n* = 3–6, *p* < 0.05; ns, not significant).

To further explore the functional implications of ATF7 in UC, chromatin immunoprecipitation sequencing (ChIP‐seq) was performed on colonic mucosal samples from patients with active UC. Analysis of the ChIP‐seq data revealed the distribution of reads around transcription start sites (TSS) (Figure 2A) and the genomic localization of enriched peaks (Figure 2B). KEGG pathway enrichment analysis of the associated genes (Figure 2C) identified several pathways, with mitophagy emerging as the pathway with the highest rich factor.

Detailed visualization of the ChIP‐seq data using the Integrative Genomics Viewer (IGV) revealed a significant enrichment of ATF7 ChIP reads at the transcription start site of PINK1, a key regulator of mitochondrial homeostasis (Figure 2D). This suggests that ATF7 may directly regulate PINK1 expression, linking it to pathways implicated in active UC.

To validate the role of ATF7 in regulating mitochondrial function, we utilized mice with intestinal epithelial cell‐specific deletion of *ATF7* and induced colitis using DSS. Transmission electron microscopy was employed to examine mitochondrial morphology in colonic epithelial cells. Compared to WT controls, ATF7‐deficient mice exhibited a substantial increase in swollen, structurally abnormal mitochondria following DSS treatment (Figure 2E). Quantitative analysis revealed a significant reduction in average mitochondrial diameter in the ^−−^ *ATF7/+* DSS group (Figure 2F), indicating increased mitochondrial damage associated with ATF7 deficiency.

These findings indicate that the loss of ATF7 exacerbates mitochondrial damage and impairs its resolution, potentially contributing to the pathogenesis of UC.

### 3.2 ATF7 Regulates PINK1‐Mediated Mitophagy and Protects Against TNF‐α‐Induced Mitochondrial Dysfunction

**FIGURE 3:**
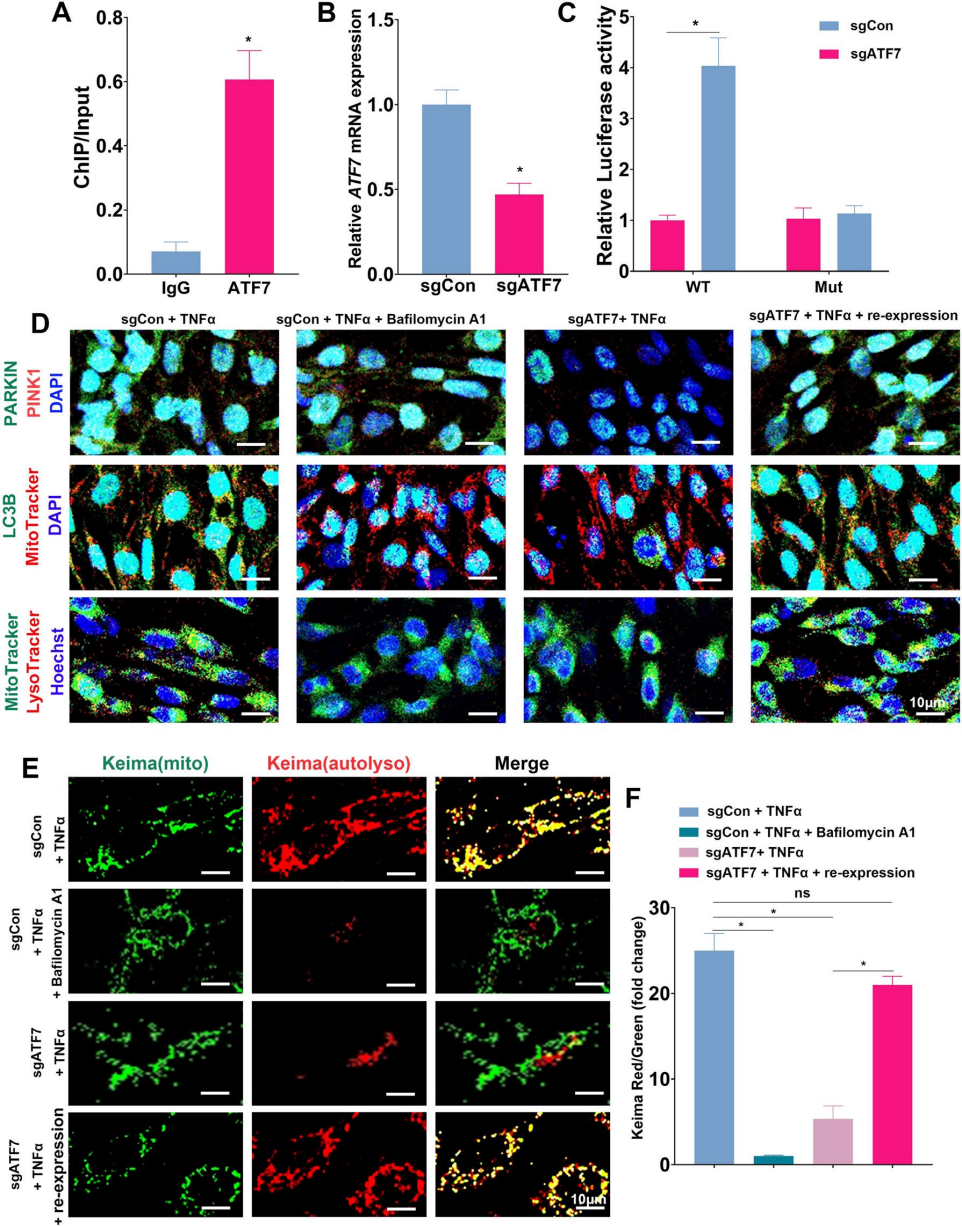
ATF7 regulates PINK1‐mediated mitophagy in intestinal epithelial cells. (A) ChIP assay showing ATF7 enrichment at the PINK1 promoter in CCD 841 CoN cells. Data are presented as ChIP/Input ratios. (B) Quantitative PCR confirming *ATF7* knockdown in CCD 841 CoN cells transfected with sgRNA targeting *ATF7* (sgATF7) compared to control sgRNA (sgCon). (C) Dual‐luciferase reporter assay showing reduced luciferase activity in cells with *ATF7* knockdown (sgATF7) using the wild‐type (WT) PINK1 promoter construct. Luciferase activity was unchanged in cells with a mutated (Mut) promoter construct. (D) Representative immunofluorescence images of CCD 841 CoN cells under the indicated conditions. Top row: Co‐localization of PARKIN (green) and PINK1 (red). Middle row: MitoTracker (red) and LC3B (green) co‐localization indicating autophagosome formation. Bottom row: Co‐localization of MitoTracker (green) and LysoTracker (red) reflecting mitolysosome formation. (E) Live‐cell imaging using the mito‐Keima probe to assess mitophagy flux. Red fluorescence (acidic environment) indicates mitolysosome formation, while green fluorescence marks mitochondria in neutral pH. (F) Quantification of red‐to‐green fluorescence ratio from mito‐Keima analysis. Data represent mean ± SD (*n* = 3–6); statistical analysis was performed using one‐way ANOVA. *p < 0.05; ns, not significant.

To further investigate the regulatory mechanism of ATF7, chromatin immunoprecipitation (ChIP) assays were performed in human intestinal epithelial CCD 841 CoN cells, identifying PINK1 as a direct target of ATF7 (Figure 3A). Subsequently, *ATF7* expression was knocked down in CCD 841 CoN cells using sgRNA, and successful knockdown was confirmed via qPCR (Figure 3B). To evaluate ATF7's role in regulating PINK1, a dual‐luciferase reporter assay was conducted. A mutation was introduced in the *PINK1* promoter and cloned into a luciferase vector. Knockdown of ATF7 led to significantly reduced luciferase activity in the wild‐type construct but not in the mutated construct, confirming that ATF7 directly regulates *PINK1* transcription (Figure 3C).

To further validate these findings, we performed in vitro experiments in CCD 841 CoN colonic epithelial cells with ATF7 knockdown under TNF‐α stimulation. To assess the functional consequences, ATF7 was re‐expressed in the knockdown cells. Western blot analysis showed that ATF7 re‐expression restored PINK1 protein levels, supporting a positive regulatory role of ATF7 in PINK1 expression (Figure S1A,B). To assess mitophagic flux, bafilomycin A1 was used to inhibit the fusion of autophagosomes with lysosomes, as recommended by current autophagy research standards [29]. Dual‐immunofluorescence analysis revealed reduced co‐localization of PINK1 and PARKIN‐key mediators of mitophagy‐i*n* ATF7‐deficient cells. Moreover, bafilomycin A1 significantly disrupted the interaction between voltage‐dependent anion channel (VDAC), a mitochondrial marker, and LC3B, an autophagosome‐associated protein. ATF7 knockdown also suppressed LC3B expression, which was restored upon ATF7 re‐expression. To further assess mitophagic flux, we examined co‐localization between MitoTracker and LysoTracker in the presence or absence of bafilomycin A1. In ATF7‐deficient cells, bafilomycin A1 led to a further reduction in MitoTracker–LysoTracker co‐localization, indicating impaired lysosomal degradation of mitochondria. This defect was reversed by ATF7 re‐expression, which also increased LysoTracker signal intensity (Figure 3D).

To strengthen these findings, Western blot analysis was conducted to evaluate the expression of key mitophagy‐related proteins, including PINK1, PARKIN, and LC3B, under TNF‐α stimulation (Figure S2). The results confirmed that ATF7 deficiency led to a marked reduction in the expression of PINK1, PARKIN, and LC3B. Notably, reexpression of ATF7 restored LC3B levels, further supporting its regulatory role in mitophagy (Figure S3).

In accordance with established autophagy guidelines [29], we employed the mito‐Keima probe to assess mitophagy flux. Both bafilomycin A1 treatment and ATF7 deficiency resulted in a marked decrease in red fluorescence, indicative of impaired mitophagic activity, whereas re‐expression of ATF7 restored red fluorescence intensity (Figure 3E,F).

**FIGURE 4:**
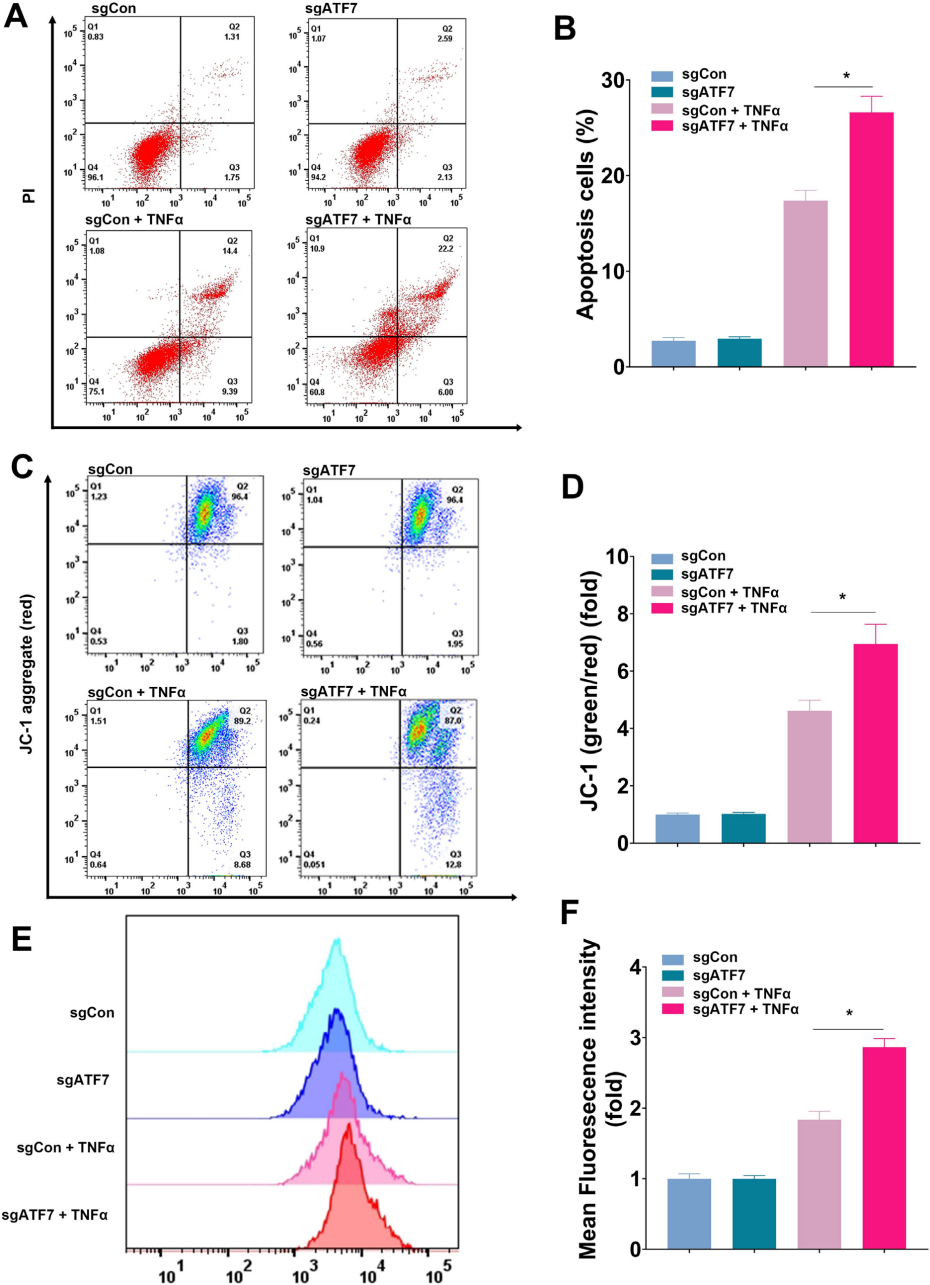
ATF7 protects against TNF‐α‐induced mitochondrial dysfunction, ROS production, and apoptosis. (A) Flow cytometry analysis of apoptotic cells using Annexin V/PI staining in control (sgCon) and *ATF7* knockdown (sgATF7) CCD 841 CoN cells with or without TNF‐α treatment. (B) Quantification of apoptotic cells (%), showing a significant increase in cell death in *ATF7*‐deficient cells upon TNF‐α stimulation. (C) Flow cytometry analysis of mitochondrial membrane potential using JC‐1 staining. A decrease in JC‐1 aggregates (red fluorescence) relative to monomers (green fluorescence) was observed in *ATF7* knockdown cells, indicating mitochondrial depolarization. (D) Quantification of JC‐1 green/red fluorescence ratio (fold change), showing exacerbated mitochondrial dysfunction in the absence of *ATF7* upon TNF‐α treatment. (E) Flow cytometry histograms of ROS levels measured using a ROS‐sensitive fluorescent dye. TNF‐α‐treated *ATF7* knockdown cells exhibited higher ROS production compared to controls. (F) Quantification of mean fluorescence intensity (fold change) confirming elevated ROS levels in ATF7‐deficient cells under TNF‐α stimulation. Data are presented as mean ± SD, with statistical significance determined by one‐way ANOVA. (*n* = 6, *p < 0.05).

The inhibition of mitophagy results in the accumulation of damaged mitochondria, elevated production of reactive oxygen species (ROS), and increased apoptosis, thereby amplifying inflammation. Flow cytometry analysis revealed a marked increase in TNF‐α‐induced cell death in *ATF7*‐deficient cells (Figure 4A,B). JC‐1 staining demonstrated a significant reduction in mitochondrial membrane potential in ATF7‐deficient cells, indicative of severe mitochondrial dysfunction (Figure 4C,D). Additionally, ATF7 deficiency significantly augmented TNF‐α‐induced ROS production, further contributing to cellular stress and damage (Figure 4E,F).

These results collectively demonstrate that ATF7 plays a crucial role in regulating PINK1‐mediated mitophagy, protecting against mitochondrial dysfunction, ROS accumulation, and cell death under inflammatory conditions.

### 3.3 ATF7 Deficiency Exacerbates DSS‐Induced Colitis Through Impaired Mitophagy and Increased ROS Production

**FIGURE 5:**
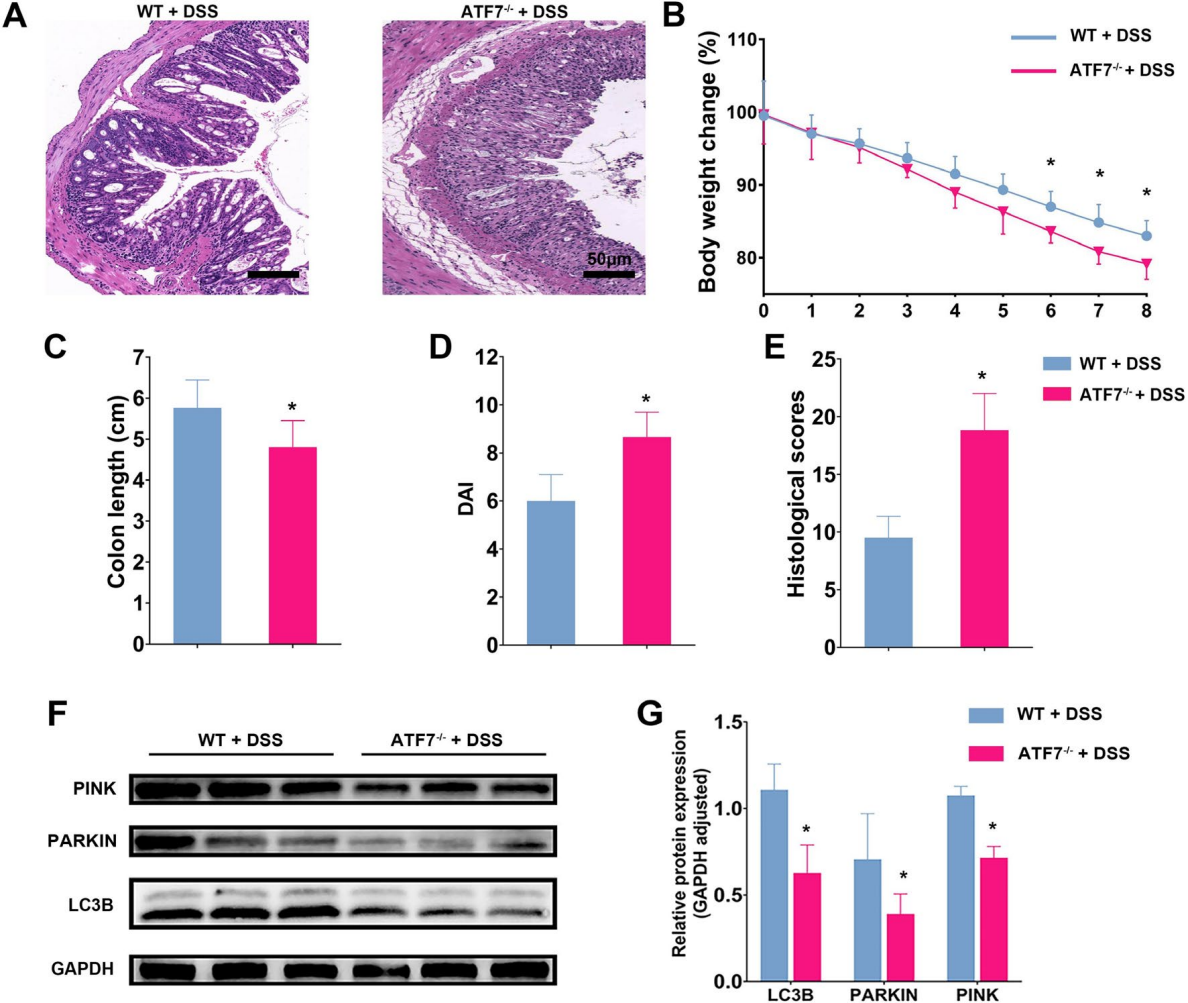
ATF7 deficiency exacerbates DSS‐induced colitis and impairs mitophagy in vivo. (A) Representative H&E‐stained colonic sections from DSS‐treated wild‐type (WT + DSS) and ATF7‐deficient (*ATF7* ^−/−^ + DSS) mice, demonstrating more severe inflammation and structural damage in ATF7‐deficient mice. (B) Percentage of body weight change during 8 days of DSS treatment, showing a greater decline in ATF7‐deficient mice. (C) Colon length measurements, indicating significant shortening in *ATF7* ^−/−^ + DSS mice. (D) Disease activity index (DAI) scores, revealing increased disease severity in *ATF7* ^−/−^ + DSS mice. (E) Histological scores reveal more severe inflammation and epithelial damage in *ATF7* ^−^/^−^ + DSS mice. (F) Immunoblot analysis of colonic tissues showing decreased expression of mitophagy‐related proteins, including PINK1, PARKIN, and LC3B, in ATF7‐deficient mice compared to WT controls. GAPDH served as a loading control. (G) Densitometric quantification of LC3B, PARKIN, and PINK1 expression normalized to GAPDH, confirming significant downregulation in *ATF7* ^−^/^−^ + DSS mice. Data are presented as mean ± SD. Statistical significance was assessed using one‐way ANOVA or unpaired two‐tailed t‐tests, as appropriate. (*n* = 6, *p < 0.05).

To further validate our in vitro findings in vivo, we utilized intestinal epithelial cell‐specific *ATF7* ^−/−^ mice and induced acute colitis using DSS. Histological analysis of colonic tissues stained with H&E revealed more severe inflammation and extensive disruption of intestinal architecture in ATF7‐deficient mice compared to wild‐type controls (Figure 5A). During DSS treatment, ATF7‐deficient mice exhibited greater body weight loss (Figure 5B), shorter colon length (Figure 5C), higher disease activity index (DAI) scores (Figure 5D), and elevated histopathological scores (Figure 5E), confirming that ATF7 deficiency exacerbates inflammation in vivo. Western blot analysis was conducted to assess the expression of mitophagy‐related proteins, including PINK1, PARKIN, and LC3B (Figure 5F). Quantitative analysis confirmed that ATF7 deficiency led to a significant reduction in the expression levels of these proteins (Figure 5G).

To assess the impact of ATF7 deficiency on colonic mitophagy, immunofluorescence staining was performed. Colocalization analysis demonstrated reduced co‐localization of PINK1 and PARKIN, critical markers of mitophagy, in ATF7‐deficient mice. Moreover, interactions between VDAC, a mitochondrial marker, and LC3B, as well as between VDAC and LAMP2, a lysosomal marker, were significantly diminished in ATF7‐deficient mice (Figure S4).

**FIGURE 6:**
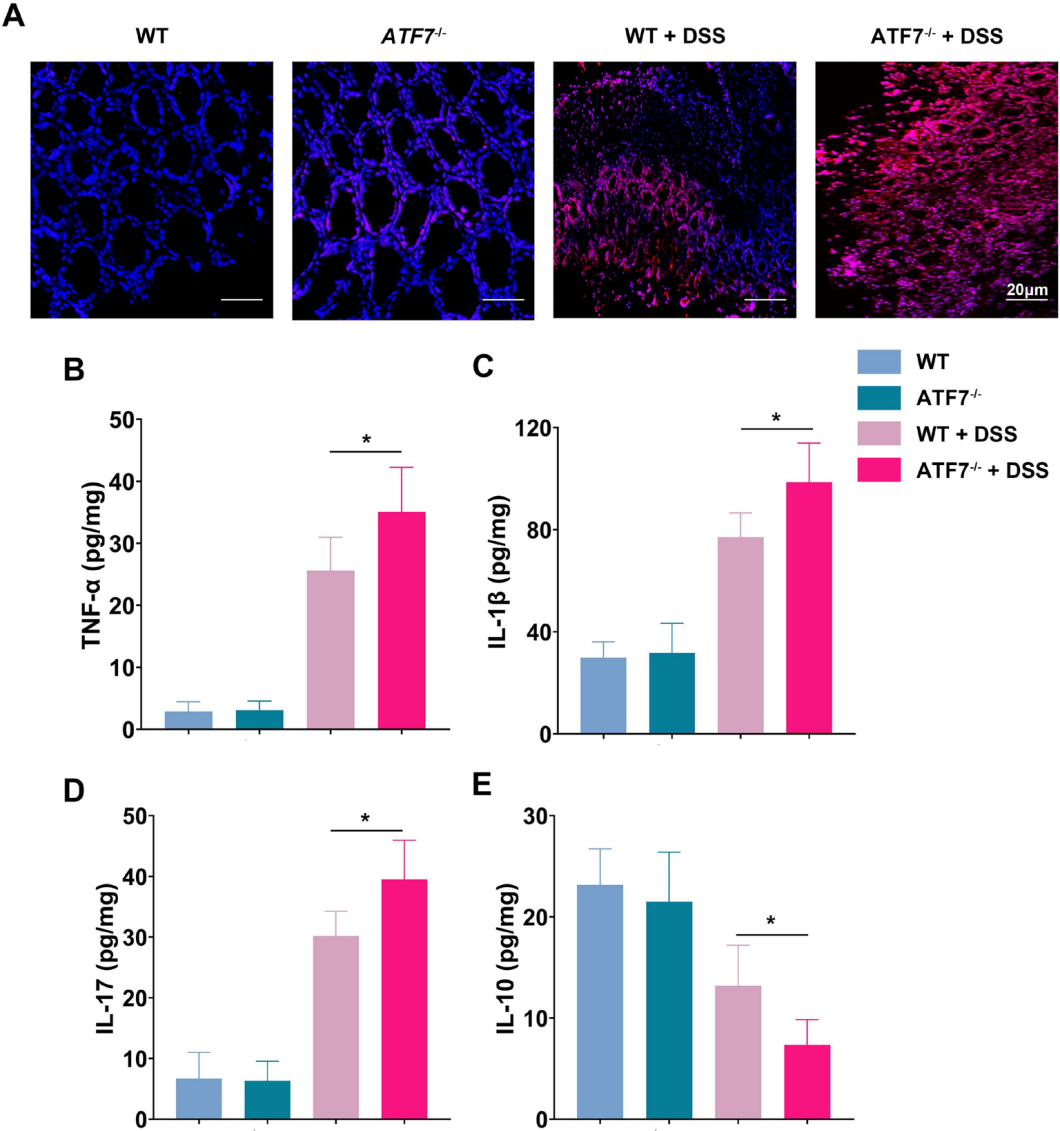
ATF7 deficiency increases ROS production and exacerbates inflammatory cytokine expression in DSS‐induced colitis. (A) Dihydroethidium (DHE) staining of colonic tissues from wild‐type (WT), DSS‐treated WT (WT + DSS), ATF7‐deficient (*ATF7* ^−/−^), and DSS‐treated ATF7‐deficient (*ATF7* ^−/−^ + DSS) mice, showing increased ROS production in *ATF7* ^−/−^ + DSS mice. (B–E) ELISA measurements of inflammatory cytokines in colonic tissues. (B) TNF‐α levels, significantly elevated in *ATF7* ^−/−^ + DSS mice compared to WT + DSS mice. (C) IL‐1β levels, showing a similar significant increase in ATF7‐deficient mice with DSS treatment. (D) IL‐17 levels, markedly higher in *ATF7* ^−/−^ + DSS mice. (E) IL‐10 levels, significantly reduced in *ATF7* ^−/−^ + DSS mice, indicating impaired anti‐inflammatory responses. Data are presented as mean ± SD, with statistical significance determined using one‐way ANOVA and unpaired two‐tailed t‐tests (*p < 0.05, *n* = 6).

The impairment of mitophagy in ATF7‐deficient mice was associated with increased intracellular ROS levels, as revealed by DHE staining of colonic tissues. Consistent with our in vitro results, ATF7 deficiency led to elevated ROS levels in DSS‐induced colitis (Figure 6A). Furthermore, ELISA analysis of colonic homogenates showed that ATF7‐deficient mice exhibited significantly increased levels of pro‐inflammatory cytokines, including TNF‐α, IL‐1β, and IL‐17, alongside a marked reduction in the anti‐inflammatory cytokine IL‐10 (Figure 6B–E).

These findings collectively demonstrate that ATF7 deficiency disrupts mitophagy, leading to ROS accumulation and heightened pro‐inflammatory responses, ultimately exacerbating intestinal inflammation in DSS‐induced colitis. This study highlights the critical role of ATF7 in maintaining mitochondrial homeostasis and mitigating inflammation in the gut.

## 4 Discussion

In our study, we have focused on elucidating the roles of Activating Transcription Factor 7 (ATF7) and PTEN‐induced kinase 1 (PINK1) within the context of ulcerative colitis (UC) and its manifestation in intestinal epithelial cells (IECs). A key finding of our research is the significant downregulation of ATF7 in the intestinal mucosa of UC patients during active disease phases (Figure 1). This reduction in ATF7 expression is closely correlated with the severity of the disease, as indicated by the Mayo score, positioning ATF7 as a potential biomarker for UC progression.

Furthering our understanding, we employed chromatin immunoprecipitation sequencing (ChIP‐seq) and dual‐luciferase assays to uncover a novel aspect of ATF7's function. We have identified that ATF7 acts as a positive regulator of *PINK1* transcription, implicating it in the process of mitophagy. This regulation is critical, as diminished ATF7 levels lead to an accumulation of damaged mitochondria, which are not efficiently cleared. Restoration of ATF7 expression in ATF7‐deficient cells followed by TNF‐α stimulation led to an upregulation of mitophagy‐associated proteins (Figures S1 and S3).

To directly assess mitophagic flux, we adopted established methodologies using mito‐Keima and bafilomycin A1 in live‐cell imaging [29]. These experiments confirmed that ATF7 re‐expression significantly enhanced mitophagy (Figure 3), underscoring its essential role in mitochondrial quality control. Impaired mitophagy due to ATF7 loss resulted in the accumulation of dysfunctional mitochondria and a consequent increase in reactive oxygen species (ROS), which in turn activated inflammatory pathways and promoted apoptosis (Figures 4 and 6). These insights not only shed light on the molecular underpinnings of UC but also open new avenues for targeted therapeutic strategies.

In the context of UC, our study has identified a significant inverse relationship between the expression of ATF7 in intestinal mucosa and the severity of the disease, as quantified by the Mayo score. This correlation suggests that ATF7 expression levels could serve as a predictive biomarker for assessing the severity of active UC. The potential of ATF7 as a biomarker is particularly compelling in the realm of personalized medicine. By evaluating ATF7 expression through biopsy samples from the intestinal mucosa, clinicians may be able to more accurately predict the course of UC in individual patients. This approach could significantly enhance the management of UC, allowing for more tailored treatment strategies based on the predicted severity of the disease.

Moreover, understanding the role of ATF7 in UC pathogenesis not only aids in prognosis but also opens new avenues for therapeutic intervention. Targeting ATF7‐related pathways could provide novel treatment options, especially in cases where traditional therapies are insufficient. The biomarker potential of ATF7, therefore, extends beyond diagnostics into the realm of treatment, offering a dual benefit in the fight against UC.

Mitochondria are well‐acknowledged as the main source of ROS in cells. The failure of mitophagy to timely clear damaged mitochondria leads to an excessive accumulation of ROS, which in turn can trigger cellular apoptosis and inflammation. Additionally, IECs are recognized as central regulators of intestinal immune homeostasis [6]. This understanding underscores the potential impact of compromised mitophagy within IECs, which could precipitate inflammation and apoptosis in these cells. Given this backdrop, our exploration of ATF7's role in regulating PINK1 expression and its subsequent involvement in mitophagy acquires heightened significance. The ATF7‐PINK1 pathway emerges not just as a mechanism for maintaining mitochondrial health but also as a critical factor in controlling the balance between cell survival and the inflammatory response in the intestinal epithelium. This insight places our study at the forefront of understanding how mitochondrial dynamics within IECs can influence the broader context of intestinal health and disease, particularly in conditions like ulcerative colitis.

Our study challenges the traditional understanding of ATF7's function, traditionally viewed as part of the ATF family involved in stress responses and autophagy regulation. While members like ATF4 are known for inducing autophagy through the regulation of genes such as Atg5, Atg7, and Atg10, ATF7's role has been less clear [35, 36]. Previously reported to activate mitophagy in *C. elegans* , playing a neuroprotective role [45], ATF7's function in mammalian systems remained understudied. Our research marks the first report of ATF7 regulating PINK1 expression and participating in mitophagy within intestinal epithelial cells, a critical insight into its role beyond stress response mechanisms (Figure 3). We demonstrate that ATF7 deficiency leads to impaired mitophagy, exacerbating colitis (Figure 5). This novel understanding of ATF7 in the context of intestinal health and disease significantly broadens our comprehension of the regulatory networks in UC. The revelation that ATF7 directly influences PINK1 expression and thereby mitochondrial integrity offers a new perspective on how cellular apoptosis and the inflammatory environment in UC are controlled.

The PINK1/Parkin pathway is one of the most well‐characterized mechanisms responsible for inducing mitophagy. In this pathway, upon mitochondrial damage or depolarization, PINK1 stabilizes on the outer mitochondrial membrane, facilitating Parkin recruitment and activation [46, 47]. Parkin ubiquitinates mitochondrial proteins, signaling their degradation. This process includes recruiting LC3, essential for directing damaged mitochondria to autophagosomes [31, 32]. These autophagosomes then fuse with lysosomes, where the damaged mitochondria are ultimately degraded, maintaining cellular homeostasis [33, 34]. Our study underscores that diminished PINK1 expression leads to a notable impairment in mitophagic activity. This is characterized by a reduced interaction between PINK1 and Parkin, a decrease in mitochondrial autophagosomes, and a subsequent reduction in mitophagic lysosomes, as detailed in Figures 3, 4, 5 of our findings. This impairment underscores the pivotal role of PINK1 in maintaining mitochondrial health and, by extension, cellular integrity.

Our findings on the ATF7‐PINK1 axis in UC unveil significant therapeutic possibilities. We demonstrate the critical role of this axis in mitochondrial health and inflammation regulation, suggesting new intervention strategies for UC. The identification of ATF7 as a key regulator in mitochondrial dynamics and inflammatory response opens avenues for targeted therapies. Enhancing ATF7 activity or modulating PINK1 may restore mitochondrial function and reduce inflammation in UC. Future research should aim to unravel the intricate interactions between ATF7 and PINK1 and their broader impact on UC, potentially extending to various cell types, including immune cells. Pharmacological strategies targeting the ATF7‐PINK1 axis, through inhibitors or activators, emerge as a promising research area. Such targeted therapies could address both mitochondrial dysfunction and inflammation, offering a refined approach to UC management. In summary, our study advocates a novel therapeutic direction in UC, focusing on the molecular interplay between ATF7 and PINK1. This approach holds promise for deepening our understanding of UC and developing innovative treatments to benefit those affected by this condition.

In concluding our study, we emphasize the significant strides made in understanding UC, focusing on the ATF7‐PINK1 axis. Our research reveals how this axis critically influences mitochondrial dynamics and inflammatory responses in IECs, offering new insights into the pathogenesis of UC. A key advancement is our identification of ATF7 as a vital regulator of mitochondrial homeostasis, closely interlinked with PINK1. This regulatory partnership is pivotal in mitigating IECs apoptosis and reducing inflammation, presenting a notable leap in our understanding of UC and its underlying molecular mechanisms.

These findings not only deepen our comprehension of UC but also suggest new directions for therapeutic intervention, targeting the ATF7‐PINK1 axis to control both cell death and inflammation. This study thereby marks a significant contribution to gastroenterology, guiding future research and potential clinical strategies in UC, and highlights the importance of targeting molecular pathways for effective disease management.

## Author Contributions

Y.C. conducted the experiments and drafted the manuscript. X.Z. and F.L. assisted with data collection. J.L. contributed to the animal experiments. Q.Y. and J.L. were involved in the literature review and data analysis. L.Z. conceptualized the study and critically revised the manuscript.

## Ethics Statement

This study was conducted in strict adherence to ethical guidelines. Ethical approval for animal experiments was obtained from the Animal Ethics Committee of Huazhong University of Science and Technology (Approval No. 2022‐3388). For human subjects, approval was granted by the Independent Ethics Committee of Wuhan Union Hospital (Approval No. 2024‐0261). The study was performed in accordance with the principles outlined in the Declaration of Helsinki.

## Conflicts of Interest

The authors declare no conflicts of interest.

## Supporting information


Data S1.

## Data Availability

All data generated or analyzed during this study are available from the corresponding author upon reasonable request.
