# Supplementary material for: ATF7–PINK1 Axis Governs Mitophagy and Intestinal Inflammation in Ulcerative Colitis
Source: FASEB J. 2025 Jun 30;39(13):e70792. doi: 10.1096/fj.202500813R (PMC12208144; doi:10.1096/fj.202500813R)
Supplement: Supplementary file 1 — Data S1. [file FSB2-39-e70792-s001.docx]

**Supplementary Table 1. Antibodies and reagents used for Western blotting and immunofluorescence staining.**

| Target Protein / Dye | Application | Supplier | Catalog Number |
| --- | --- | --- | --- |
| GAPDH | Western blot | Cell Signaling Technology | 5174S |
| ATF7 | Western blot | Abcam | ab183507 |
| PINK1 | Western blot / Immunofluorescence | Abcam | ab216144 |
| PARKIN | Western blot | Abcam | ab77924 |
| PARKIN | Immunofluorescence | Abcam | ab15494 |
| LC3B | Western blot / Immunofluorescence | Sigma-Aldrich | L7543 |
| VDAC | Immunofluorescence | Abcam | ab14734 |
| LAMP2 | Immunofluorescence | Abcam | ab125068 |
| MitoTracker Red | Mitochondrial labeling | Thermo Fisher Scientific | M7512 |
| MitoTracker Green | Mitochondrial labeling | Thermo Fisher Scientific | M7514 |
| LysoTracker Red | Lysosomal labeling | Thermo Fisher Scientific | L7528 |

**Supplementary Figure. 1**

**
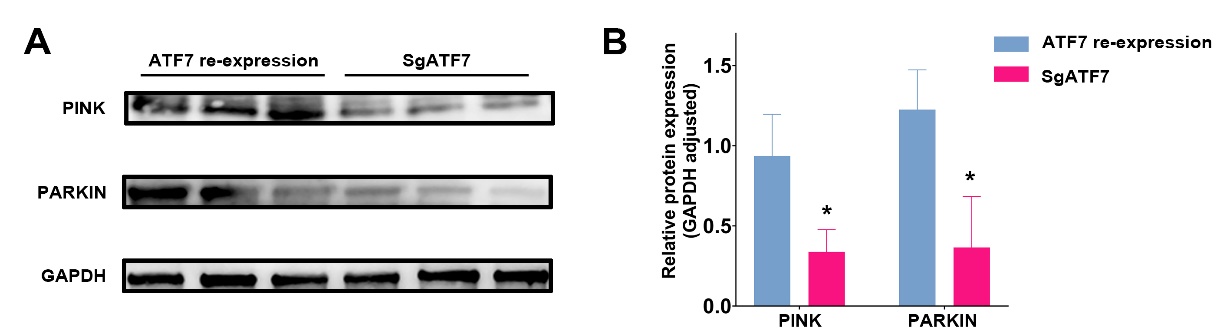
**

**Supplementary Figure. 2**

**
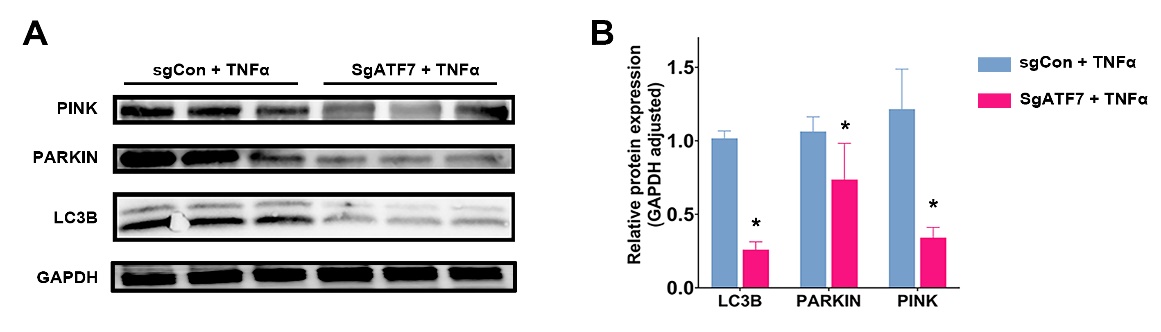
**

**Supplementary Figure. 3**

**
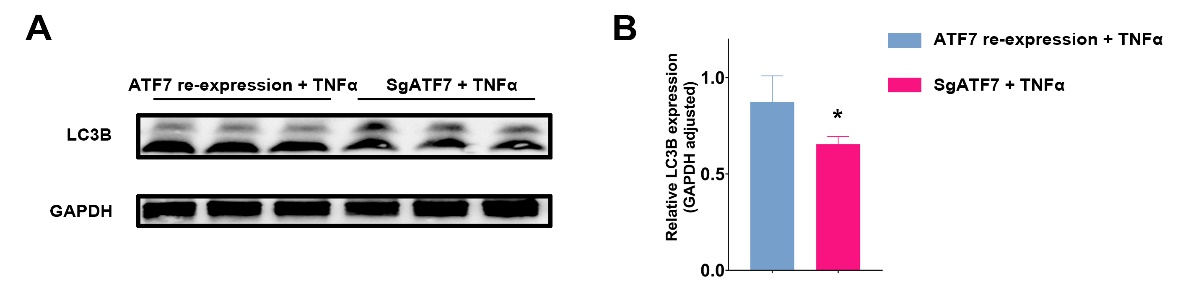
**

**Supplementary Figure. 4**

**
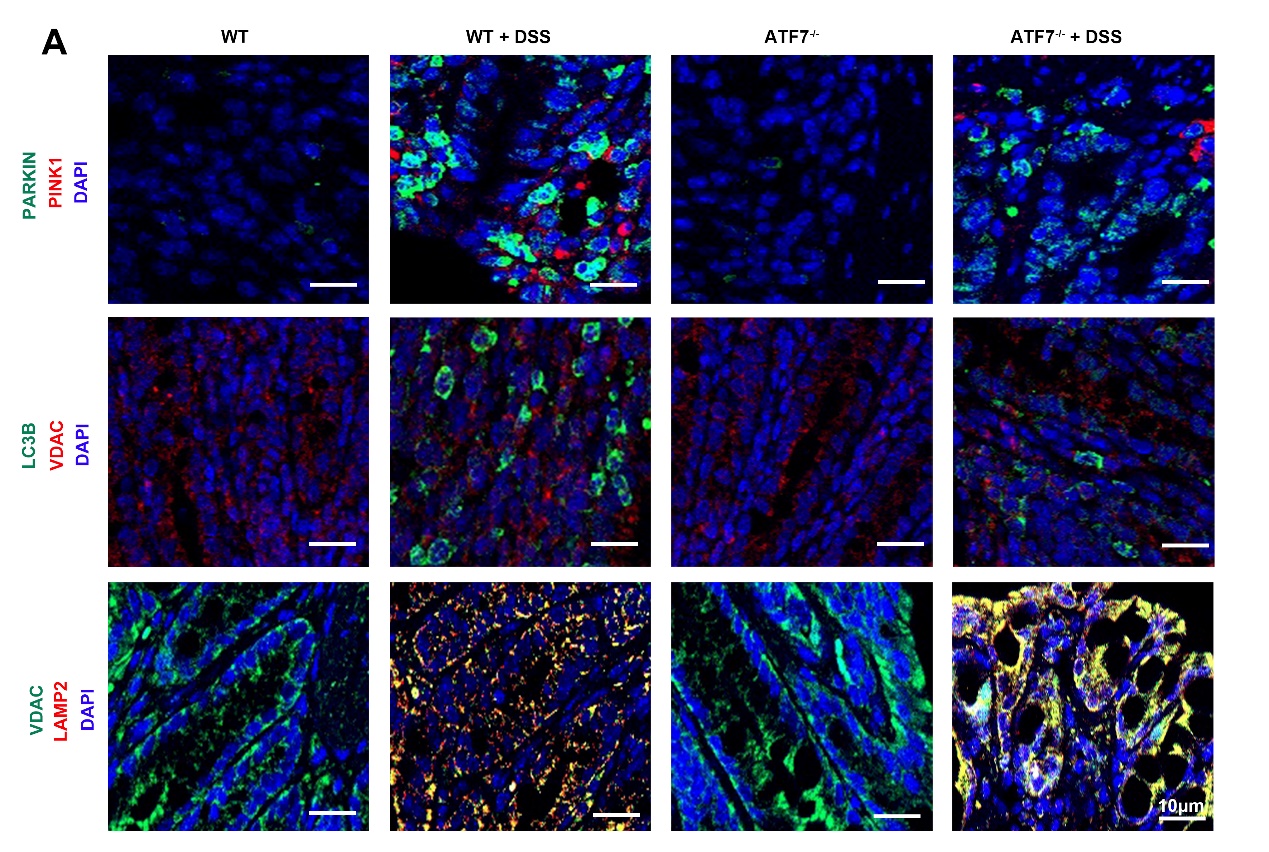
**

**Supplementary Figure. 1. ATF7 re-expression restores PINK1 and PARKIN levels in ATF7-deficient colonic epithelial cells.**

(A) Representative Western blot analysis of PINK1 and PARKIN protein expression in CCD 841 CoN cells following ATF7 knockdown (SgATF7) or re-expression. GAPDH served as a loading control. (B) Densitometric quantification of PINK1 and PARKIN protein levels normalized to GAPDH. Data are shown as mean ± SD. (**P* < 0.05, unpaired t-test, n = 6).

**Supplementary Figure 2. Western blot analysis of mitophagy-related protein expression in vitro.**

(A) Representative Western blot images showing the expression of PINK1, PARKIN, LC3B, and GAPDH in CCD 841 CoN cells with ATF7 knockdown under TNF-α induction. (B) Quantification of PINK1, PARKIN, and LC3B protein levels in (A), demonstrating a significant reduction in protein expression in ATF7-deficient cells compared to controls. Data are presented as mean ± SD, with statistical significance determined by one-way ANOVA (n = 6, **P* < 0.05).

**Supplementary Figure. 3. ATF7 re-expression restores LC3B expression under TNF-α stimulation in ATF7-deficient intestinal epithelial cells.**

(A) Western blot analysis of LC3B protein levels in CCD 841 CoN cells subjected to ATF7 knockdown (SgATF7) or re-expression, following TNF-α treatment. GAPDH was used as a loading control. (B) Quantification of LC3B expression normalized to GAPDH. Data represent mean ± SD. (n = 6, **P* < 0.05).

**Supplementary Figure 4. ATF7 deficiency impairs colonic mitophagy as evidenced by reduced colocalization of key mitophagy markers.**

Representative immunofluorescence images of colonic sections from wild-type (WT), DSS-treated WT (WT + DSS), *ATF7*⁻/⁻, and DSS-treated *ATF7*⁻/⁻ (*ATF7*⁻/⁻ + DSS) mice. Top row: Co-staining of PINK1 (red) and PARKIN (green) shows decreased colocalization in ATF7-deficient mice, indicating impaired mitophagy initiation. Middle row: Co-staining of LC3B (green) and VDAC (red), a mitochondrial outer membrane marker, reveals reduced colocalization in *ATF7*⁻/⁻ groups, reflecting defective autophagosome-mitochondria interactions. Bottom row: Co-staining of LAMP2 (red), a lysosomal marker, and VDAC (green) shows attenuated lysosome-mitochondria fusion in the absence of ATF7. Nuclei were counterstained with DAPI (blue). (n = 6).
